# Plasmid Profiles and Prevalence of Intermediately Virulent *Rhodococcus equi* from Pigs in Nakhonpathom Province, Thailand: Identification of a New Variant of the 70-kb Virulence Plasmid, Type 18

**DOI:** 10.4061/2010/491624

**Published:** 2009-12-09

**Authors:** Chaithep Poolkhet, Suksun Chumsing, Worawidh Wajjwalku, Chihiro Minato, Yukiko Otsu, Shinji Takai

**Affiliations:** ^1^Faculty of Veterinary Medicine, Kasetsart University, Nakhonpathom 73140, Thailand; ^2^School of Veterinary Medicine and Animal Science, Kitasato University, Aomori 034-8628, Japan

## Abstract

The prevalence of intermediately virulent *Rhodococcus equi* isolates from pig submaxillary lymph nodes from four slaughterhouses in Nakhonpathom province, Thailand, was investigated. The isolates were tested for the presence of virulence plasmids and the 20-kDa virulence-associated protein antigen (VapB) gene by PCR. Of the 734 submaxillary lymph nodes tested, 19 (2.6%) produced positive cultures of *R. equi*. All 19 isolates were positive for the VapB gene and contained virulence plasmids that were identified as type 1 (six isolates), type 6 (two isolates), type 7 (one isolate), type 16 (two isolates), and a new variant (eight isolates). Based on the restriction digestion patterns of the plasmid DNAs, we tentatively designated the variant as type 18. Investigation of the prevalence and plasmid profiles of VapB-positive *R. equi* in pigs should be extended throughout Thailand to evaluate potential sources of zoonotic infections.

## 1. Introduction

The actinomycete *Rhodococcus equi *is a facultative, intracellular, Gram-positive coccobacillus bacterium. Three types of *R. equi *have been classified as virulent, intermediately virulent, and avirulent on the basis of their virulence-associated protein (Vap) antigens and virulence plasmids [1–3]. Virulent *R. equi *is characterized by the presence of the 15–17-kDa virulence-associated protein antigen (VapA) and virulence plasmids of 85–90 kb and is the cause of pyogranulomatous bronchopneumonia in foals [1]. Previous studies have identified at least 12 different virulence plasmids in VapA-positive *R. equi* [4]. Intermediately virulent *R. equi *is characterized by the presence of a 20-kDa virulence-associated protein antigen (VapB) and virulence plasmids of 79–100 kb and can be found in pig submaxillary lymph nodes and immunocompromised patients [5]. At this time, 27 different plasmids have been identified in VapB-positive *R. equi *[6]. Intermediately virulent (VapB-positive) *R. equi *has been reported to cause fatal cavitary pneumonia because of secondary infections in immunocompromised hosts, such as AIDS patients [7, 8]. Avirulent *R. equi *is widespread in the soil environment and is isolated predominantly from immunocompromised patients without AIDS [9]. 

 In Thailand, infection by *R. equi *has been reported in patients with and without HIV infection in Chiang Mai, and 52 isolates from 69 sporadic cases collected between 1993 and 2001 were VapB-positive *R. equi *[10, 11]. Isolates of VapB-positive *R. equi *collected from healthy pigs at an abattoir and from immunodeficient patients in Chiang Mai contained the same plasmid types [11], so the pig was thought to be a source of VapB-positive *R. equi *infections in immunocompromised patients [5, 10, 11]. A study to examine the plasmid pattern of VapB-positive *R. equi* to evaluate any correlation between human infection and pig infection in each area was necessary to prove the source of infection. This study aimed to improve our knowledge of intermediately virulent *R. equi* in Thailand by investigating the prevalence and plasmid types of VapB-positive *R. equi* in the submaxillary lymph nodes of pigs in the central Thailand province of Nakhonpathom.

## 2. Materials and Methods

### 2.1. Bacterial Strains

Seventeen representative strains of intermediate virulence (human and pig origins: Table 1) were used as plasmid-type reference strains. Some of the protein profiles, plasmid characteristics, and virulence levels of these strains have been described previously [5, 7, 10, 11].

### 2.2. Collection of Lymph Nodes from Slaughterhouses

Submaxillary lymph nodes were removed from freshly slaughtered pigs at 4 slaughterhouses in Nakhonpathom province, Thailand, from June 2004 to October 2005, and placed in sterile dishes for transport to the laboratory.

### 2.3. Isolation and Identification of Intermediately Virulent *R. equi*

The lymph nodes were immersed in boiling water for 3 seconds before they were cut up finely with sterile scissors. The pieces were placed onto nalidixic acid- novobiocin-actidione (cycloheximide)-potassium tellurite (NANAT) selective medium plates, as described previously [12]. The plates were incubated at 38°C in an incubator for two or three days. All suspected colonies of *R. equi *were counted, and several colonies per specimen was subcultured and then identified in our laboratory (CAMP test positive, catalase positive, urease positive, nitrate reduction test positive; glucose, sucrose, maltose, and esculin were not fermented).

### 2.4. Isolation and Examination of Plasmid DNA

Plasmid DNA was extracted from the isolates by the modified alkaline lysis method, as described previously [5, 13]. Plasmid DNA samples were digested with restriction endonucleases *Eco*RI and *Eco*T221, and the plasmid samples were separated on 1.0% agarose gels at approximately 5 V/cm for 2 hours, and compared with reference plasmids.

### 2.5. Polymerase Chain Reaction

The target gene for polymerase chain reaction (PCR) amplification was the published sequence of the VapB gene (GenBank D44469) from *R. equi *strain 5. The primers were 5′-GACTCTTCACAAGACGGT-3′ and 5′-TAGGCGTTGTGCCAGCT-3′ for the sense and antisense strands, respectively [14]. PCR amplification was performed using 20 *μ*L of the DNA preparation in a 100 *μ*L reaction that contained 20 mM Tris-HCl (pH 8.3 at 25°C), 100 mM KCl, 2.5 mM MgCl2, 0.4 mM of each dNTP, 1 *μ*M of each primer, and 2.5 U of Taq DNA polymerase (Takara, Tokyo, Japan), as described previously, with some modifications [11]. The samples were amplified for 35 cycles on a thermocycler (BioRad) under the following conditions: denaturation for 90 seconds at 94°C, annealing for 1 minute at 55°C, and extension for 2 minutes at 72°C [2].

## 3. Results and Discussion

### 3.1. Results

VapB-positive *R. equi *was isolated from 19 (2.6%) of 734 pig submaxillary lymph nodes collected from four slaughterhouses (prevalence range, 0% to 6.3%) in Nakhonpathom province. All were confirmed to be VapB-positive *R. equi *by biochemical tests and PCR amplification of the VapB gene. The plasmid profiles of the 19 isolates were investigated by restriction enzyme digestion with EcoRI and EcoT221. The 19 isolates contained virulence plasmids identified as type 1 (six isolates, 0.8%), type 6 (two isolates, 0.3%), type 7 (one isolate, 0.1%), type 16 (two isolates, 0.3%), and a new variant (eight isolates, 1.1%).

**Figure 1:**
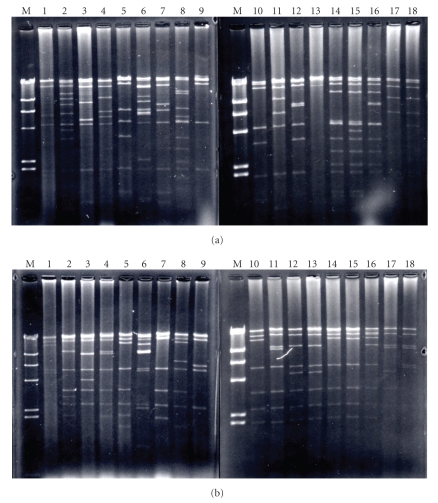
*Eco*RI (a) and *Eco*T22I (b) restriction fragments of the plasmid types of *R. equi* isolates of intermediate virulence. Lanes 1, strain A2 (plasmid type 1); lanes 2, strain S2 (plasmid type 2); lanes 3, strain S3 (plasmid type 3); lanes 4, strain S4 (plasmid type 4); lanes 5, strain A5 (plasmid type 5); lanes 6, strain S6 (plasmid type 6); lanes 7, strain S7 (plasmid type 7); lanes 8, strain S8 (plasmid type 8); lanes 9, strain S9 (plasmid type 9); lanes 10, strain A11 (plasmid type 10); lanes 11, strain A43 (plasmid type 11); lanes 12, strain 70 (plasmid type 12); lanes 13, strain H3 (plasmid type 13); lanes 14, strain H25 (plasmid type 14); lanes 15, strain H43 (plasmid type 15); lanes 16, strain H66 (plasmid type 16); lanes 17, strain 316 (plasmid type 17); lanes 18, new strain (plasmid type 18). The markers (lanes M) are *Hind*III digestion products of bacteriophage *λ* DNA.

The DNA of the 17 representative plasmids and the one new type was digested with *Eco*T22I and examined by Southern analysis with PCR probes. The PCR products labeled with digoxigenin-11-dUTP hybridized with one of the fragments of each plasmid DNA. From these results, we tentatively designated the new plasmid type 18. Based on the restriction digestion patterns of the plasmid DNA of the new variant, we estimated the size of the new variant to be approximately 70 kb (Figure 1).

### 3.2. Discussion

This study has shown that pigs bred in Nakhonpathom province, in the central part of Thailand, have VapB-positive *R. equi *in their submaxillary lymph nodes, with a prevalence of 2.6%, which is similar to that observed in Japan [5]. Previous studies demonstrated that *R. equi *was widespread (average prevalence 14%) in the submaxillary lymph nodes of domestic and wild pigs in Hungary and that the majority of *R. equi *strains from pigs were avirulent; only 25.6%–26.8% of the isolates in these studies were intermediately virulent (VapB-positive) and none were virulent (VapA-positive) *R. equi* [6, 7]. The prevalence of intermediately virulent *R. equi *in Hungarian pigs was quite different from that demonstrated in this study or in our previous study, which showed that 368 (93.9%) of 392 isolates collected from the submaxillary lymph nodes of Japanese pigs were intermediately virulent, whereas two (0.5%) of the isolates were virulent and the remaining 22 (5.6%) were avirulent [5]. Differences in the breeding of pigs in Hungary (pigs are kept in the natural environment), Thailand (pigs are kept in the backyards of farmers' houses), and Japan (pigs are kept on large-scale farms) may reflect the differences in the prevalence of each type of *R. equi* in pig isolates among these three countries.

 A new variant was found in eight isolates from pigs collected at one of four slaughterhouses. Before this study, at least 18 distinct 70–100-kb plasmids that are associated with the expression of VapB have been identified in pig and human isolates of *R. equi *[2, 5, 6, 11, 15, 16]. However, during our study, other new types of VapB-positive have been reported by Makrai et al. (2008) [6]. Previous studies show that the immunocompromised individuals were at particular risk of infection with zoonotic diseases. *R. equi *is an emerging pathogen of humans, particularly in those with a compromised immune system [17]. Our recent studies in Thailand and Hungary have shown that the infecting *R. equi *strains are more closely related to isolates from regional pigs than other animals [7, 10, 11]. Munsakul et al. reported two cases of pulmonary rhodococcosis in AIDS patients in Bangkok. There was no information concerning the source or route of infection in those two patients, such as contact with pigs or other domestic animals [18]. So, exploring geographic difference between AIDS patient's infection and pig's isolation in plasmid profiles of intermediately virulent *R. equi* may be a useful molecular fingerprint for epidemiological purposes to prove a route of infection in human. 

 The pathogenicities of these representative strains in pigs are interesting, but we do not have enough information on the pathology of the submaxillary lymph nodes of the pigs from which VapB-positive *R. equi *has been isolated. Komijn et al. (2007) investigated the prevalence of granulomatous lesions in the lymph nodes of about two million pigs in the Netherlands and isolated *R. equi *from 44 of 98 (44.9%) submaxillary lymph nodes with granulomatous lesions [19]. Experimental infections in pigs showed no clinical signs other than transient fever and weight loss. Intermediately virulent strains were recovered in cultures from various organs, and the lymph nodes of pigs inoculated intravenously, but only from the mandibular lymph nodes of pigs inoculated intramuscularly [20].

## 4. Conclusions

This study showed the distribution of the plasmid profiles of VapB-positive *R. equi *isolates from healthy pigs from slaughterhouses in Nakhonpathom province, Thailand, and described a new plasmid type, type 18. This is the first report of the prevalence of VapB-positive *R. equi *in pigs in the central part of Thailand.

## Figures and Tables

**Table 1:** The reference strains of intermediately virulent *R. equi* with human and pig origins were used in this study.

Plasmid types	Number of reference strains
1	A2
2	S2
3	S3
4	S4
5	A5
6	S6
7	S7
8	S8
9	S9
10	A11
11	A43
12	70
13	H3
14	H25
15	H43
16	H66
17	316
